# Intravestibular Stapes Prosthesis Protrusion Causing Post Stapedectomy Vertigo

**DOI:** 10.5334/jbr-btr.951

**Published:** 2015-12-30

**Authors:** J. P. Toirkens, W. P. A. Kelders

**Affiliations:** 1 Radiology department, Havenziekenhuis, Haringvliet 2, 3011 TD, Rotterdam, The Netherlands; 2 ENT department, Havenziekenhuis, Haringvliet 2, 3011 TD, Rotterdam, The Netherlands

**Keywords:** stapes prosthesis, vertigo

## Abstract

Post stapedectomy vertigo is most often a self-limiting postoperative complication. Sometimes vertigo occurs years after operation and different etiologies, non-surgically as well as related to previous surgery, have to be excluded. High resolution CT of the temporal bone can be of help, as in this case report, in which the images showed intravestibular stapes prosthesis protrusion.

## Case report

A 49-year-old woman complains of new onset episodes of vertigo post stapedectomy 12 years ago. Symptoms can be evoked by pressing or when lying on the left ear. No remarkable findings on ear inspection or clinical exam. Epley maneuvers had no effect for treatment of possible benign paroxysmal positional vertigo (BPPV). Other differential diagnoses were perilymphatic fistula, intravestibular granuloma, labyrinthitis and stapes prosthesis protrusion.

**Figure 1:**
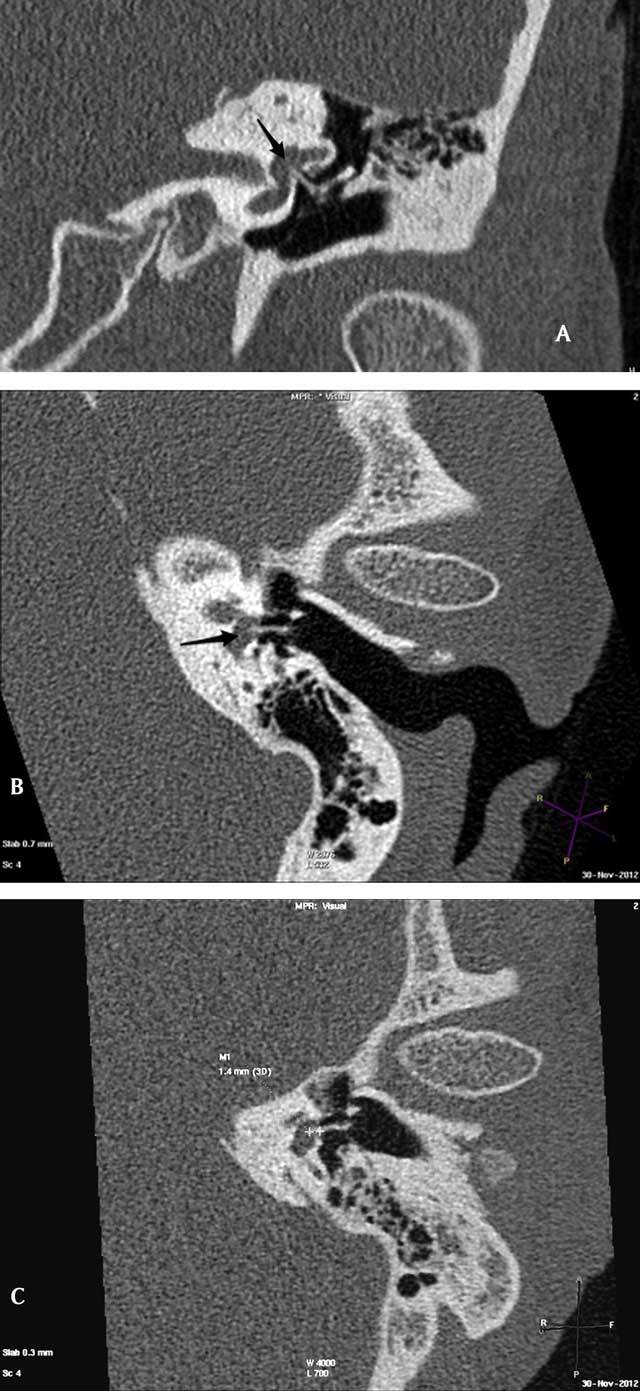
A) Para-coronal; B) Para-axial multiplanar reformats show stapes prosthesis protruding into the vestibule (arrow); C) measurement of protrusion depth.

A temporal bone high resolution CT (HR CT) was performed and showed migration of the stapes prosthesis into the vestibule (protrusion) (Fig. 1). Depth of protrusion was measured 14 millimeters from tip of the prosthesis to the oval window. The diagnosis of intravestibular stapes prosthesis protrusion related post stapedectomy vertigo was made and revision surgery was proposed.

## Discussion

Stapes surgery is performed to restore hearing in patients frequently because of underlying otosclerosis. Other reasons can be posttraumatic ossicular reconstruction, cicatricial adhesions or tympanosclerosis.

Stapedectomy and more recently stapedotomy are the most frequent types of surgery in which the otosclerotic disease causing hearing loss is overcome by removing part of the footplate or drilling a hole in de footplate after removing the stapes superstructure leaving the footplate in place (stapedotomy). Ossicular reconstruction is then often realized by introducing a piston like prosthesis that extends from the lenticular process of the incus to the footplate.

Over 84–90% of patients benefit from surgery [1,2,3], but 0.2–3% can suffer from sensorineural hearing loss and/or vertigo postoperatively [4]. Prolonged vertigo can be caused by stapes prosthesis protrusion into the vestibule. Supposed underlying pathophysiological mechanism is pressure on the vestibular organs, especially the macula of the utricle, and thereby causing inadvertent rotational sensation. Otological evaluation of these patients is often difficult.

HR CT imaging can frequently help to find the cause of the vertigo [5]. HR CT can demonstrate the position of the stapes prosthesis tip in relation to the oval window. 0.5–1 millimeter is the generally accepted limit for normal vestibular penetration [6,7]. This limit has been established in cadaver studies and is related to the minimal distance from the stapes footplate in stapedotomy to the vestibular organs. Caution has to be taken not to make harsh conclusions out of near limit CT measurements because small cadaver case control studies have shown that precise measurement of the stapes protrusion until recently were not possible. Maybe last generation multislice CT systems can narrow the gap between multiplanar measurement and reality. Also the prosthesis material could have an effect on measurements (metal vs. plastic). Nevertheless HR CT can be of great use differentiating between possible other causes of post stapes surgery vertigo like granuloma (local soft tissue mass) or perilymphatic fistula (not visible).

One of the most frequent causes of positional vertigo, BPPV, has to be taken into account. For this easy clinical tests and positional therapy like Epley maneuver can be used (patient has to sit on side of bed and turn his/her head 45 degrees, then quickly turn to supine position with a 30 degree neck extension and keep this position for 1–2 min, then turn his/her head 90 degrees to the other side, wait another 1–2 min and then slowly return to upright position and wait 30 sec).

These findings can help to decide which patients might have to be reoperated on.

In conclusion intravestibular protrusion of stapes prosthesis can be a cause of late onset post-stapedectomy/stapedotomy vertigo, which can be detected with HR CT.

## Competing Interests

The authors declare that they have no competing interests.
